# Online health information seeking and the association with anxiety among older adults

**DOI:** 10.3389/fpubh.2023.1076571

**Published:** 2023-02-10

**Authors:** Amy M. Schuster, Reza Ghaiumy Anaraky, Shelia R. Cotten

**Affiliations:** ^1^Department of Sociology, Anthropology and Criminal Justice, Clemson University, Clemson, SC, United States; ^2^Department of Technology Management and Innovation, Tandon School of Engineering, New York University, New York City, NY, United States; ^3^Department of Communication, Clemson University, Clemson, SC, United States

**Keywords:** online health information seeking, older adults, NHATS, longitudinal, anxiety

## Abstract

**Introduction:**

The Internet supplies users with endless access to a wealth of information and is generally the first source searched by U.S. adults (18 years and older) when seeking health information. Age and anxiety are associated with online health information seeking (OHIS). Older adults (65 years and older) are increasing their OHIS. Importantly, OHIS can potentially lead to improved health outcomes for older adults. The relationship between OHIS and anxiety is less clear. Studies report those with more symptoms of anxiety are more likely to be OHIS, while other studies find the reverse pattern or no association. Generalized anxiety disorder affects up to 11% of older adults and is oftentimes unrecognized and untreated.

**Methods:**

To address the mixed findings in the literature, we analyzed six waves (2015–2020) of data from the National Health and Aging Trends Study to assess the causal relationship between anxiety and OHIS using a Random Intercept Cross-lagged Panel Model framework.

**Results:**

We found that while anxiety symptoms lead to OHIS in the next wave, OHIS was not associated with anxiety symptoms in the next wave.

**Discussion:**

This suggests that for this sample of older adults, OHIS does not reduce or exacerbate older adults' symptoms of anxiety.

## Introduction

The growth of the Internet has provided users unlimited access to an abundance of information, including health information. The Internet is typically the first source searched by U.S. adults (18 years and older) when seeking health information (1), in order to alleviate uncertainty and increase medical understanding (2). Online health information seeking (OHIS) is mainly performed to find personal health information, request personal health information (e.g., test results, appointments), or to find health information for others (3).

There has been a steady increase in the percentage of Internet using older adults who are OHIS (4), from 14.5% in 2011 to 43.6% in 2020 (5). In addition, the COVID-19 pandemic prompted a rapid increase in the number of older adults using the Internet to access health information (6, 7). Importantly, OHIS increases older adults' health information knowledge which aides them in decision-making related to health concerns (8). OHIS gives older adults a feeling of empowerment in managing their health and has been associated with increased ability to provide self-care and increased health-related quality of life (8, 9).

Older adults are heterogenous in terms of their OHIS (10). They use different devices, such as smartphones or tablets, to access health information (11). Young-old older adults (65–74 years), compared to those older, are more likely to be OHIS (4, 12, 13) and to use social media for health-related information (14). For example, 63% of community-dwelling older adults, 66–74 years old, use the Internet to search for health information; this number drops to 49% for community-dwelling older adults aged 75 years and older (15). In addition to age, gender, education, annual income, and health status influence older adults' level of engagement with OHIS. Older adults who are female and who have more chronic health symptoms are more likely to be OHIS (16–20), whereas older adults with less than a high school education and an annual household income of < $25,000 are less likely to be OHIS (4).

Though research focusing on older adults' OHIS is increasing, less is known about how OHIS is related to well-being outcomes among older adults, such as anxiety. On average, 15% of older adults have anxiety symptoms (21) such as constant worrying, being on edge, fearful, or having a hard time concentrating. Having anxiety symptoms is not the same as having an anxiety disorder. Diagnostic criteria for anxiety disorders include having extreme, persistent worry and fear that is difficult to control and affects daily tasks, social life, and relationships (22). Up to 14% of older adults meet the diagnostic criteria for an anxiety disorder (23). Among older adults, generalized anxiety disorder (GAD) is the most common type of anxiety disorder with a prevalence rate of up to 11% (24–26). GAD is characterized by chronic, excessive feelings of dread about routine life issues, activities, or events, where the worst is anticipated, although there is little reason for this belief (27). GAD is often accompanied by physical symptoms such as being easily fatigued, unexplained pains (chest, head, muscles), trembling, sweating, cognitive decline, and feeling out of breath (27, 28). Among older adults, GAD is often unrecognized and untreated, as anxiety symptoms may be unacknowledged or ignored by the older adult or are overlooked by medical professionals who consider the physiological symptoms related to normal aging-related changes (28). Untreated anxiety disorders can decrease older adults' ability to perform daily activities, reduce their overall health, and lower their quality of life (28).

GAD is influenced by a range of social, economic, and genetic factors (29). Research has found that older adults with GAD are excessively concerned about health-related issues, finances, family problems, or the potential for disaster, and they may have problems sleeping, focusing, and relaxing (28). Interestingly, OHIS has been associated with a decrease in anxiety symptoms (3). Most of the research related to OHIS and anxiety has concentrated on health anxiety, the fear of having a critical medical condition even though they have minimal or no symptoms [see articles by Kontos et al. (19) and Berle et al. (20)], yet < 10% of older adults experience health anxiety (30–33). However, the relationship between OHIS and anxiety is not well understood at this time. This could be due to studies using different research designs, samples, OHIS and anxiety measures, and analytic approaches. Coglianese et al. (3) performed a longitudinal cohort study analyzing 2 days of data for 105 Italian hospitalized pregnant women using the 40-item State-Trait Anxiety Inventory and the 3-part UIH questionnaire on online information-seeking behaviors. They reported that OHIS and anxiety had a negative association. Berle et al. (20) used a cross-sectional design and sampled 992 adults from Australia, Canada, Ireland, New Zealand, the United Kingdom, and the U.S. using the 7-item PROMIS-Emotional Distress Anxiety Short Form Scale and a 1-item OHIS measure. The found no association between anxiety symptoms and OHIS.

Five studies on the relationship between OHIS and anxiety had samples that included older adults. Cotten et al. (34) analyzed data from the National Cancer Institute's 2005 Health Information National Trends Survey with 2,929 internet using adults (18 years and older) using two measures (amount of online activities created by summing 10 OHIS activities and the 6-item K6 scale). They reported that more OHIS activities (5 or more) were significantly associated with more symptoms of distress. They posit that when individuals seek online health information, they may find varying information, some of which may be inaccurate or misleading. As individuals try to navigate this information, it may lead to uncertainty, which could increase anxiety levels and psychological distress. When individuals experience anxiety and/or distress because of their OHIS, it may lead to further OHIS in an effort to reduce uncertainty. Thus, higher anxiety might be associated with higher levels of OHIS. Conversely, if the information that is found reduces anxiety, it may reduce further OHIS. Choi and DiNitto (35) analyzed one wave (2011) of the National Health and Aging Trends Study data which uses a 2-item Generalized Anxiety Disorder scale (GAD-2) and the 4-item Internet use for health-related tasks measure. They found that higher anxiety symptoms were associated with less OHIS for older adults (65 years and older). de Looper et al. (36) employed a pre/post-test design using the 6-item State-Trait Anxiety Inventory and a 1-item OHIS measure with a sample aged 33–88 years. They reported that OHIS was not significantly related to feelings of anxiety before or after a cancer consultation. Myrick and Willoughby (37) analyzed data from the 2013 Health Information National Trends Survey (HINTS 4, Cycle 3) which used a 1-item anxiety measure and a 1-item OHIS measure and found that U.S. adults (*M* = 54.68 years, SD = 16.47) who reported feeling anxious all the time were significantly less likely to go online and search for health information. Seçkin (38) used a cross-sectional design with a 1-item adverse effect measure and multiple OHIS measures and reported that U.S. adults (18–93 years, *M* = 48.82, SD = 16.43) who used Internet health information to manage their healthcare (e.g., self-diagnose, self-treat) had more feelings of worry and anxiety. Due to variability in research designs we cannot determine if OHIS is affecting feelings of anxiety or vice versa. It is likely that feelings of anxiety could lead to OHIS and that OHIS could also lead to feelings of anxiety.

To address the gaps in the literature and the dearth of longitudinal studies examining the relationship between OHIS and GAD among older adults, this study focused on the symptoms of anxiety with a longitudinal sample consisting of only older adults (65 years and older). Though we are not testing a formal theory, we build upon work by Cotten et al. (34) who discussed potential pathways through which ICT use affects mental health. In our case, we speculate that OHIS can help older adults make informed life decisions. However, given that older adults are more likely to experience health concerns, it may be the case that finding more information online may exacerbate anxiety, particularly if they do not have medical professionals to discuss information with or if the information they find online suggests a more serious health concern. Conversely, if the information they find online assuages their worries, it may lessen anxiety. This study is guided by the research question, how do OHIS and anxiety impact each other over time? Based on the literature, we hypothesize that (1) anxiety symptoms will have a negative and longitudinal impact on OHIS and (2) OHIS will have a positive and longitudinal impact on symptoms of anxiety.

## Materials and methods

### Study sample

We used data from wave 5 (2015) to wave 10 (2020) of the National Health and Aging Trends Study (NHATS) which is comprised of a nationally representative sample of older adults (age 65 and above). The NHATS collects information annually through in-person interviews with older adults on their mental and physical function to investigate late in life trends. Data collection began in 2011, with a sample of 8,245 older adults; however, due to participant dropouts or death, the number of respondents decreased each year. To address this sample change, a new cohort of 4,182 participants was added in 2015, creating a total of 8,334 participants in wave 5. For this study, we included 6 waves (2015–2020) of data given: (1) the sample was updated in wave 5 (2015), and (2) the 2020 data was the last available data at the time we carried out the analysis. The baseline in our study was defined at wave 5. We excluded proxy responses and those residing in a nursing home (1,277 participants). The final sample size for this study was 7,057 older adults.

### Measures

#### Online health information seeking

In the NHATS, older adults were asked if they use the Internet or not, *In the last month have you ever gone on the Internet or online for any reason?* Those who respond “yes” were then prompted to answer more detailed questions about their Internet use. For this study, OHIS was measured with three Internet use questions*: In the last year, have you gone on the Internet or online to: (1) contact any of your medical providers, (2) handle Medicare or other insurance matters, (3) get information about your health conditions* (35, 39–41). Response options included yes (coded as 1) or no (coded as 0) to each of these questions. We used a sum score, ranging from 0 to 3, to operationalize OHIS. A higher score reflected more OHIS. Cronbach's alpha for OHIS was 0.651.

#### Generalized anxiety disorder

Anxiety was assessed through the 2-item Generalized Anxiety Disorder scale (GAD-2): *Over the last month, (1) how often have you felt nervous, anxious, or on the edge? (2) how often have you been unable to stop or control worrying?* (42). The response options ranged from 0 = “not at all” to 3 = “nearly every day.” To operationalize anxiety, responses were summed, ranging from 0 to 6. A higher score indicated more anxiety symptoms. The GAD-2 is a validated brief screening measure for assessing anxiety symptoms with good psychometric properties (cut off point ≥3: 86% sensitivity, 83% specificity) (42, 43). NHATs adapted the measure from a 2 week to a month time frame. Cronbach's alpha for GAD was 0.644.

### Covariates

We controlled for the following sociodemographic information (age, education, gender, race, living arrangement) and health status [limitations in activities of daily living (ADLs) and self-reported health]. Age (65–107 years) was measured as a continuous variable. Education was measured with eight categories, including “no school”, “1–12th grades”, “high school diploma”, “trade school diploma”, “some college”, “associate degree”, “bachelor's degree”, and “graduate degree.” Since some levels of education did not have many observations, treating it as a categorical variable was not feasible; therefore, we treated it as a continuous variable. Gender [1 (male), 0 (female)], race [White (reference category), Black, Other], and living arrangement [0 living alone (never married, widowed, or divorced), 1 living with partner (living with partner or married)] were dummy coded. Race, gender, and level of education were analyzed at baseline (2015). Living arrangement was analyzed at each given wave. In the cases where the living arrangement data was missing in a given wave, we used the last existing data. Limitations in ADLs were measured through four questions measuring the extent of difficulty respondents experienced in using the toilet, eating, getting dressed, and showering or washing up by themselves and without help during the last month. The responses were recorded by a 4-item Likert scale of none, a little, some, or a lot. We used the sum score of these four measures as limitations in ADLs, ranging from 4–16. A self-reported health variable was measured by asking the following question: “Would you say that, in general, your health is excellent, very good, good, fair, or poor?” Self-reported health ranged from 1–5, with a higher score signifing better health.

### Analytic strategy

We used a Random Intercept Cross-lagged Panel Model (RI-CLPM) framework to assess the causal relationship between anxiety symptoms and OHIS. The RI-CLPM includes auto-regressive and cross-lagged effects (44). Autoregressive effects allow the state of a unit to be a function of its past state. Cross-lagged effects assess whether one variable at time t can predict another variable at time *t* + 1. The RI-CLPM is a popular method for scholars to study causal relationships between two variables using longitudinal data (3, 20, 36, 37). Furthermore, we followed Hamaker et al. (44) approach by conducting both unconstrained and constrained models. In the unconstrained model, the auto-regressive and cross-lagged effects can vary from wave to wave. In the constrained model, however, model specification constrains the auto-regressive and the cross-lagged effects to be equal over time. The superior model is determined by comparing the fit indices between the unconstrained and constrained models. If the two models are not significantly different, the model is time non-invariant and we choose the constrained model (i.e., the more parsimonious model). If the unconstrained model fits significantly better than the constrained model, it means that the auto-regressive and lagged effects vary among waves. In this case, we proceeded with the unconstrained model. We used the sample weights on 2015 (the baseline year) provided by the NHATS dataset for the analysis. Although the NHATS data has sample weights for each wave, we used a wide format in the RI-CLPM analysis and were only able to use one analytic sample weight. All the analyses were carried out in R v.4.2. We used the Lavaan package and a full-information maximum likelihood estimator to conduct the RI-CLPM analyses (45).

## Results

Older adults in this study at baseline were, on average, aged 77.76 years (SD = 7.62) and were predominantly White (68.97%) and female (57.50%). Table 1 reports participants' demographic information. At baseline, OHIS was on average 0.84 (SD = 0.99) and anxiety was 0.82 (SD = 1.27).

**Table 1:** Sample demographics.

	**2015**	**2016**	**2017**	**2018**	**2019**	**2020**
	**(*****N** =* **7,057)**	**(*****N** =* **5,897)**	**(*****N** =* **5,161)**	**(*****N** =* **4,589)**	**(*****N** =* **4,111)**	**(*****N** =* **3,524)**
	***N*** **(%)**					
**Gender**						
Female	4,060 (57.50)	3,406 (55.76)	2,969 (57.53)	2,650 (57.75)	2,384 (57.99)	2,042 (57.95)
Male	2,997 (42.50)	2,491 (42.24)	2,192 (42.47)	1,939 (42.25)	1,727 (42.01)	1,482 (42.05)
**Race**						
White	4,867 (68.97)	4,150 (70.37)	3,633 (70.39)	3,255 (70.93)	2,926 (71.17)	2,553 (72.44)
Black	1,431 (20.28)	1,177 (19.95)	1,045 (20.24)	913(19.89)	817 (19.87)	689 (19.55)
Other	759 (10.76)	570 (9.66)	483 (9.35)	421 (9.17)	368 (8.95)	282 (8.80)
Living with partner	3,489 (49.40)	2,877 (48.78)	2,475 (47.95)	2,171 (47.30)	1,915 (46.58)	1,631 (46.28)
	***M*** **(SD)**					
Age	77.76 (7.62)	78.46 (7.44)	79.10 (7.20)	79.74 (6.99)	80.34 (6.79)	80.75 (6.48)
Education	4.06 (1.73)	4.08 (1.73)	4.10 (1.73)	1.73 (4.12)	4.13 (1.73)	4.18 (1.74)
Overall health	3.23 (1.05)	2.72 (1.53)	2.36 (1.68)	2.10 (1.75)	1.86 (1.75)	1.63 (1.77)
Limitations in ADLs	0.33 (0.76)	0.34 (0.78)	0.36 (0.80)	0.37 (0.81)	0.41 (0.86)	0.40 (0.86)
Anxiety	0.82 (1.27)	0.81 (1.26)	0.80 (1.28)	0.77 (1.23)	0.76 (1.22)	0.79 (1.23)
OHIS	0.84 (0.99)	0.91 (1.04)	0.95 (1.06)	0.96 (1.07)	1.06 (1.06)	1.21 (1.10)

N's vary depending on the level of missing data across the variables.

We conducted a RI-CLPM to assess the causal relationships between anxiety and OHIS. We controlled for age, gender, race, living arrangement, education, overall health, and limitations in ADLs. First, we conducted the unconstrained model. This model fit the data good (CFI = 0.969, TLI = 0.951, RMSEA = 0.016, 90% CI: [0.015, 0.017], SRMR = 0.042, $\chi_{\left(217\right)}^{2}$ = 618.417, *p* < 0.001) (46, 47). Next, we conducted a constrained model where the auto-regressive and the cross-lagged parameters were equal across the waves. Similar to the unconstrained model, the model fit indices suggested a good fit for the constrained model (CFI = 0.968, TLI = 0.959, RMSEA = 0.015, 90% CI: [0.014, 0.016], SRMR = 0.043, $\chi_{\left(265\right)}^{2}$ = 667.187, *p* < 0.001) (46). We followed the criteria recommended by Chen (48) to compare the two models. The criteria suggests accepting time invariance when ΔCFI < 0.010, ΔRMSEA < 0.015, and ΔSRMR < 0.030. The comparisons of our model fit indices were far away from the cutoff points suggested by Chen and the two models are not significantly different from one another. Therefore, we could infer that the model was time invariant, and proceeded with the constrained (more parsimonious) model. We report only the constrained model.

**Figure 1:**
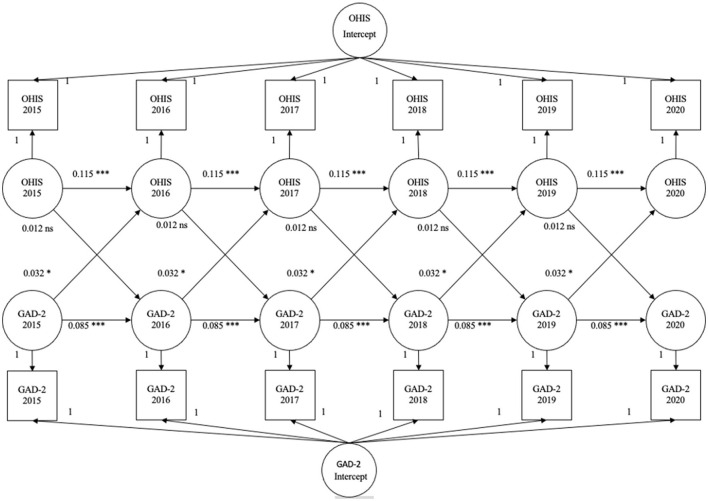
Results of RI-CLPM. *p < 0.05, ***p < 0.001, ns = not significant.

Controlling for age, gender, race, marital status, level of education, limitations in ADLs, and self-reported health, our results suggest a cross-lagged effect of anxiety on OHIS such that a higher level of anxiety would lead to having a higher OHIS in the consequent waves (β = 0.032, *p* = 0.023). Hypothesis 1 was rejected. In addition, OHIS did not predict anxiety in the next wave (β = 0.012, *p* = 0.563). Hypothesis 2 was rejected. In addition, we found two within-person autoregressive effects. Anxiety at a given wave predicted anxiety in the next wave (β = 0.085, *p* < 0.001). Likewise, OHIS at a given wave predicted OHIS in the next wave (β = 0.115, *p* < 0.001). On average, this model accounted for 17.26 percent of the variance in anxiety and 1.45 percent of the variance in OHIS. Figure 1 shows these results. To keep Figure 1 readable, we did not include information about the control variables. Table 2 presents the results, including the control variables, and how they predict the dependent variables.

**Table 2:** The results of the RI-CLPM.

**DV: Anxiety**									
	**2015**			**2016**			**2017**		
	β	**SE**	***p*** **-value**	β	**SE**	***p*** **-value**	β	**SE**	***p*** **-value**
**Between-level**									
Age	−0.009	0.003	< 0.001	−0.006	0.003	0.032	−0.004	0.003	0.173
Gender (vs. Female)	−0.311	0.036	< 0.001	−0.306	0.038	< 0.001	−0.297	0.040	< 0.001
Race (Black vs. White)	−0.167	0.044	< 0.001	−0.145	0.046	0.002	−0.155	0.050	0.002
Race (Other vs. White)	0.023	0.062	0.716	0.004	0.067	0.955	−0.033	0.069	0.627
Education	−0.035	0.010	0.001	−0.039	0.011	< 0.001	−0.033	0.011	0.004
Living with partner (vs. alone)	−0.065	0.038	0.088	−0.025	0.038	0.519	0.041	0.040	0.306
**Within-level**									
Limitations in ADLs	0.236	0.017	< 0.001	0.236	0.017	< 0.001	0.236	0.017	< 0.001
Overall health	−0.231	0.011	< 0.001	−0.231	0.011	< 0.001	−0.231	0.011	< 0.001
Anxiety (prior wave)	–	–	–	0.085	0.016	< 0.001	0.085	0.016	< 0.001
OHIS (prior wave)	–	–	–	0.012	0.020	0.563	0.012	0.020	0.563
	**2018**			**2019**			**2020**		
	β	**SE**	***p*** **-value**	β	**SE**	***p*** **-value**	β	**SE**	***p*** **-value**
**Between-level**									
Age	−0.002	0.003	0.423	−0.005	0.003	0.125	−0.005	0.003	0.114
Gender (vs. Female)	−0.285	0.040	< 0.001	−0.281	0.042	< 0.001	−0.396	0.045	< 0.001
Race (Black vs. White)	−0.056	0.052	0.282	−0.181	0.050	< 0.001	−0.107	0.055	0.053
Race (Other vs. White)	0.054	0.072	0.450	0.016	0.072	0.818	0.042	0.078	0.594
Education	−0.025	0.011	0.028	−0.048	0.012	< 0.001	−0.020	0.012	0.105
Living with partner (vs. alone)	0.022	0.041	0.593	0.002	0.043	0.958	0.093	0.046	0.043
**Within-level**									
Limitations in ADLs	0.236	0.017	< 0.001	0.236	0.017	< 0.001	0.236	0.017	< 0.001
Overall health	−0.231	0.011	< 0.001	−0.231	0.011	< 0.001	−0.231	0.011	< 0.001
Anxiety (prior wave)	0.085	0.016	< 0.001	0.085	0.016	< 0.001	0.085	0.016	< 0.001
OHIS (prior wave)	0.012	0.020	0.563	0.012	0.020	0.563	0.012	0.020	0.56348pt
**DV: OHIS**									
	**2015**			**2016**			**2017**		
	β	**SE**	***p*** **-value**	β	**SE**	***p*** **-value**	β	**SE**	***p*** **-value**
**Between-level**									
Age	−0.012	0.003	< 0.001	−0.017	0.004	< 0.001	−0.017	0.004	< 0.001
Gender (vs. Female)	0.046	0.043	0.284	0.076	0.046	0.099	−0.008	0.048	0.863
Race (Black vs. White)	−0.197	0.058	0.001	−0.169	0.068	0.013	−0.120	0.072	0.094
Race (Other vs. White)	−0.228	0.069	0.001	−0.097	0.081	0.231	0.001	0.098	0.994
Education	0.098	0.011	< 0.001	0.098	0.012	< 0.001	0.099	0.013	< 0.001
Living with partner (vs. alone)	0.129	0.042	0.002	0.132	0.045	0.004	0.152	0.046	0.001
**Within-level**									
Limitations in ADLs	0.017	0.017	0.319	0.017	0.017	0.319	0.017	0.017	0.319
Overall health	−0.012	0.012	0.318	−0.012	0.012	0.318	−0.012	0.012	0.318
Anxiety (prior wave)	–	–	–	0.032	0.014	0.023	0.032	0.014	0.023
OHIS (prior wave)	–	–	–	0.115	0.020	< 0.001	0.115	0.020	< 0.001
	**2018**			**2019**			**2020**		
	β	**SE**	**p** **-value**	β	**SE**	**p** **-value**	β	**SE**	**p** **-value**
**Between-level**									
Age	−0.019	0.004	< 0.001	−0.021	0.004	< 0.001	−0.023	0.004	< 0.001
Gender (vs. female)	0.043	0.050	0.393	0.094	0.051	0.064	0.110	0.053	0.040
Race (Black vs. White)	−0.124	0.076	0.102	−0.208	0.080	0.009	−0.006	0.088	0.947
Race (Other vs. White)	−0.204	0.093	0.028	−0.272	0.096	0.005	0.096	0.116	0.410
Education	0.114	0.013	< 0.001	0.095	0.014	< 0.001	0.075	0.015	< 0.001
Living with partner (vs. alone)	0.137	0.048	0.004	0.007	0.048	0.885	0.056	0.052	0.281
**Within-level**									
Limitations in ADLs	0.017	0.017	0.319	0.017	0.017	0.319	0.017	0.017	0.319
Overall health	−0.012	0.012	0.318	−0.012	0.012	0.318	−0.012	0.012	0.318
Anxiety (prior wave)	0.032	0.014	0.023	0.032	0.014	0.023	0.032	0.014	0.023
OHIS (prior wave)	0.115	0.020	< 0.001	0.115	0.020	< 0.001	0.115	0.020	< 0.001

Overall, males had lower levels of anxiety compared to females (*p*s < 0.001). Males also showed higher OHIS behaviors in wave 2020 (*p* < 0.05). However, in the other waves, their OHIS was slightly higher, but not significantly different from females. Among the older adults, those who were older showed slightly lower levels of anxiety in waves 2015 and 2016 and less OHIS behaviors across all the waves (*p*s < 0.001). Living with a partner (vs. alone) led to higher levels of anxiety only in wave 2020 (*p* < 0.05) and higher OHIS in waves 2015 to 2018 (*p*s < 0.01). Black respondents reported lower anxiety than White respondents in waves 2015, 2016, 2017, and 2019 (*p*s < 0.05). Furthermore, Black respondents (in waves 2015, 2016, and 2019) and respondents of other races (in waves 2015, 2018, 2019) showed less OHIS behaviors (*p*s < 0.05). Those with a higher education reported lower levels of anxiety (in waves 2015 to 2019, *p*s < 0.05) and higher OHIS behaviors (across all the waves, *p*s < 0.001).

At the within-level, an increase in one's limitations in ADLs was associated with an increase in one's level of anxiety (*p* < 0.001) but not any difference in OHIS behaviors (*p* > 0.05). Furthermore, a deterioration in one's overall perceived health led to having higher levels of anxiety (*p* < 0.001), but not any difference in OHIS behaviors (*p* > 0.05).

## Discussion

This study advances research on the relationship between OHIS and anxiety in two ways. We examined the longitudinal reciprocal relationship between OHIS and anxiety, as the results regarding the relationship between OHIS and anxiety have been mixed in prior studies (3, 20, 36, 37). We also examined these relationships specifically among older adults, a group for which little is known about OHIS and anxiety (34–38).

For our main research objective, examining the causal relationship between OHIS and anxiety, our results suggest that while older adults' feelings of anxiety drive their OHIS, OHIS does not significantly affect anxiety symptoms. This could be because older adults searching online for health information can spring from their anxiety as they strive to find information regarding their own health or that of their social ties. It is likely that limitations in ADLs and perceived health status are related to these patterns given that each was related to higher levels of anxiety in our analysis. Further research is needed with more precise health indicators to better discern the nature of these relationships.

While anxiety leads to more OHIS, the reverse was not found. OHIS was not related to changes in anxiety. It may be the case that older adults' interactions with technology and the feedback they receive from it may not be able to mitigate anxiety. Yet, it also does not exacerbate anxiety. OHIS may affect other aspects of well-being (34), which were not examined in the current study. In addition, research is also needed to further investigate older adults' perceptions of the health information they find online, whether it is perceived as credible, and how they choose to use this information, which have been shown to be important for other demographic groups (18).

Future studies should explore this scenario further and investigate older adults' expectations that may not be fulfilled by the current OHIS procedures, and whether and how technology can adapt to older adults needs to better meet their expectations. In particular, technology developers should focus on providing interface designs that facilitate easy use for older adults and those who may not be as skilled in using technologies for OHIS. Being able to easily find reliable and valid online health information and to easily identify that the information is reliable and valid may help mitigate anxiety. Conversely, trouble finding this information or in knowing whether the information is reliable and valid could potentially exacerbate anxiety. Revisiting the technology design and providing a better OHIS experience for older adults can benefit them in several ways. First, an OHIS procedure tailored to older adults' needs can potentially encourage more older adults to search for online health information. In addition, while current OHIS practices do not mitigate older adults' anxiety, an older-adult-friendly OHIS procedure can potentially mitigate some of older adults' anxiety. This may indirectly improve older adults' quality of life by reducing their anxiety (28).

Among our sample of Internet using older adults, OHIS increased slightly with each wave. This result is consistent with the literature reporting a steady increase over the years of older adults who are OHIS (15). In addition, we found that across each wave, young-old older adults were more likely to be OHIS, which is also consistent with the literature (4, 16–18). Researchers also need to be cognizant of rapid changes, like COVID-19, that create new opportunities and new challenges for older adults to search for health resources *via* the Internet. During the COVID-19 pandemic, there was a rapid increase in the number of older adults who were OHIS for information and using online portals to communicate with providers (6, 49, 50), which suggests that researchers who analyze future waves of NHATS data may find additional pathways through which OHIS and anxiety are interrelated.

## Limitations

There are a few limitations to note in this study. First, the NHATS dataset is comprised of self-reported responses so there is the possibility of recall bias. Second, NHATS has a dichotomized measure for the different types of Internet use in the past year which limited the depth of the OHIS measure used in this study. Interestingly, none of the previous cross-sectional studies on the relationship between OHIS and anxiety reported the same results as our study which could be related to the variability of OHIS measures used in each study. Three studies measured OHIS with 1-item but different questions (20, 36, 37). Two studies had more thorough measures that included questions related to type of health information searched and frequency of search (3, 38). This could also be due to the difference in research designs between the studies, with our study being longitudinal and focusing only on older adults. OHIS is not a linear process or a single event (51, 52). Research is needed that utilizes longitudinal data that includes more robust OHIS measures which assess the amount of use, type of use, reasons for the use, and how the health information was used. Knowing these aspects of OHIS may help to further elucidate how anxiety and OHIS are interrelated among older adults. Third, due to the timeframe change for the GAD-2 from 2-weeks to a month the reliability of the measure decreased. However, a lower Cronbach's alpha is not uncommon with a 2-item measure (53) and for NHATS measures where a timeframe has been adapted (54).

## Practical implications and future directions

In conclusion, this study suggests that a causal relationship exists between anxiety and OHIS, with higher anxiety leading to more OHIS among this sample of older adults. It does not suggest, however, that OHIS is bad for older adults in terms of inducing or exacerbating anxiety. Nor does OHIS reduce anxiety. Further research using more nuanced measures of OHIS may provide further delineation of the impacts of OHIS on older adults' well-being. While this study suggests that OHIS does not affect anxiety level in older adults in this sample, further longitudinal research utilizing more robust measures of OHIS are clearly warranted.

## Data availability statement

Publicly available datasets were analyzed in this study. This data can be found here: https://nhats.org/researcher/nhats.

## Ethics statement

NHATS was approved by the Johns Hopkins University Institutional Review Board and all participants provided informed consent. The patients/participants provided their written informed consent to participate in this study.

## Author contributions

SC conceived the project and provided feedback on and edited the first draft. RG performed the analysis and together with AS wrote the first draft of the manuscript. All authors revised and approved the final version of the manuscript.
